# Relationships between clinician-level attributes and fidelity-consistent and fidelity-inconsistent modifications to an evidence-based psychotherapy

**DOI:** 10.1186/s13012-015-0308-z

**Published:** 2015-08-13

**Authors:** Shannon Wiltsey Stirman, Cassidy A Gutner, Paul Crits-Christoph, Julie Edmunds, Arthur C. Evans, Rinad S. Beidas

**Affiliations:** 1 National Center for PTSD, VA Boston Healthcare System, and Boston University School of Medicine, Department of Psychiatry, 150 S. Huntington Ave (116B3), Boston, MA 02130 USA; 2 Center for Psychotherapy Research, Department of Psychiatry, University of Pennsylvania Perelman School of Medicine, 3535 Market Street, Philadelphia, Philadelphia USA; 3 Department of Psychiatry, Harvard University Medical School, Boston, MA USA; 4 Philadelphia Department of Behavioral Health and Developmental disAbility Services, 3535 Market Street, Philadelphia, USA; 5 Center for Mental Health Policy and Services Research, Department of Psychiatry, University of Pennsylvania Perelman School of Medicine, 3535 Market Street, Philadelphia, USA

## Abstract

**Background:**

Clinicians often modify evidence-based psychotherapies (EBPs) when delivering them in routine care settings. There has been little study of factors associated with or implications of modifications to EBP protocols. This paper differentiates between fidelity-consistent and fidelity-inconsistent modifications and it examines the potential influence of two clinician characteristics, training outcomes, and attitudes toward EBPs on fidelity-consistent and fidelity-inconsistent modifications of cognitive behavioral therapy in a sample of clinicians who had been trained to deliver these treatments for children or adults.

**Methods:**

Survey and coded interview data collected 2 years after completion of training programs in cognitive behavioral therapy were used to examine associations between successful or unsuccessful completion of training, clinician attitudes, and modifications. Modifications endorsed by clinicians were categorized as fidelity-consistent or fidelity-inconsistent and entered as outcomes into separate regression models, with training success and attitudes entered as independent variables.

**Results:**

Successful completion of a training program was associated with subsequent fidelity-inconsistent modifications but not fidelity-consistent modifications. Therapists who reported greater openness to using EBPs prior to training reported more fidelity-consistent modifications at follow-up, and those who reported greater willingness to adopt EBPs if they found them appealing were more likely to make fidelity-inconsistent modifications.

**Conclusions:**

Implications of these findings for training, implementation, EBP sustainment, and future studies are discussed. Research on contextual and protocol-related factors that may impact decisions to modify EBPs will be an important future direction of study to complement to this research.

The past decade has seen an increase in efforts to implement evidence-based psychotherapies (EBPs) and psychosocial interventions in public sector mental health settings [1]. Considerable attention has been devoted to factors that influence the implementation and sustainability of EBPs [2–4]. Little is known about the long-term use of EBPs after implementation, but the few studies that have examined the issue have found that fewer than 50 % of clinicians deliver them with fidelity following training [5, 6]. Additionally, consistent with implementation frameworks [7, 8], clinicians report making modifications, defined as changes to the content or method of delivery that are not specified in the standard treatment protocol, to EBPs when delivering them in routine care settings [9–11].

Different modifications can occur in routine care settings, and distinguishing between types of modifications may be useful both conceptually and for the purpose of understanding whether different kinds of modifications have implications for clinical or implementation outcomes. As Fig. 1 illustrates, the concept of modification includes adaptation. Adaptation has been defined as a process of thoughtful and deliberate alteration to the design or delivery of an intervention, with the goal of improving its fit or effectiveness in a given context [12, 13]. Typically, adaptation includes a plan to monitor the impact of the adaptation. However, clinicians also make modifications in the absence of a process that is consistent with planned adaptation [9, 11]. Such modification can occur in response to unanticipated challenges that arise in a given session or context.

**Fig. 1:**
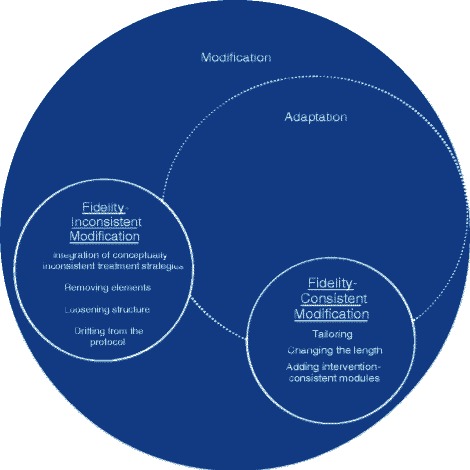
Conceptualization of modification and adaptation. Note: Modification is defined as a change to the content or method of EBP delivery that is not specified in the standard treatment protocol. Adaptation is a form of modification characterized by thoughtful and deliberate alteration to the design or delivery of an intervention, with the goal of improving its fit or effectiveness in a given context, and which includes a plan to monitor the impact of the adaptation

In conceptualizing modifications to evidence-based mental health treatments, it is important to consider the relationship between modification and treatment fidelity. Fidelity has been described as comprising adherence to the intervention components, competence with which the intervention is delivered, and differentiation from other treatments [14, 15]. Implementation frameworks suggest an important difference between modification of core elements of the intervention and changes made to less essential, peripheral components [7]. We therefore define fidelity-consistent modifications as those which do not alter core elements of treatment significantly enough to reduce adherence to a protocol and do not reduce ability to differentiate between treatments. We consider fidelity-inconsistent modifications to be those that reduce or preclude the delivery of core elements or decrease ability to differentiate between treatments. Because the goal of planned adaptation includes preservation of core intervention elements [12, 13], as Fig. 1 illustrates, fidelity-inconsistent modifications are less likely to be planned adaptations than are fidelity-consistent modifications, although there may be times when it is determined that removal of core elements or integration of other evidence-based elements are indicated, based on available data and clinical feedback.

A framework of modifications has been developed to further specify the types of changes that can be made to a protocol [16], including the following:

- Adding new, intervention-consistent elements that were not originally included in the intervention (e.g., adding a new module on a topic not originally covered in a protocol but using the same general approach to addressing the topic)
- Removing intervention components (e.g., not delivering a component of the intervention that is specified in the protocol)
- Integrating components of other established interventions (e.g., delivering components of another intervention in addition to the protocol elements)
- Tailoring aspects of the intervention (such as language, terminology, or explanations of intervention components) for an individual patient or population (e.g., simplifying worksheets or handouts while retaining key elements of the intervention)
- Loosening the structured aspects of the intervention (e.g., allowing structured, specified element of treatments such as check-ins, agendas, and end-of-session feedback to occur but in a less formal or more fluid manner)
- Changing the length of the intervention without adding or removing aspects of the intervention (e.g., extending the number of sessions without adding elements to the protocol)
- Temporary drift or deviation from the core elements of the intervention as originally designed (e.g., briefly stopping the intervention and intervening in another way before returning to the protocol)

Some of these modifications can be considered inherently fidelity-consistent or fidelity-inconsistent based on several considerations, including implementation frameworks that differentiate between core and peripheral elements of interventions [6, 7], the elements of fidelity (adherence to core intervention elements as specified in fidelity rating forms or identified through research to be necessary to produce desired clinical outcomes, competence, and treatment differentiation) [14], and empirical findings regarding modifications that may result in diminished clinical outcomes [17]. Based on these factors, we identified modifications in the framework that could be made without altering the content of core elements of the intervention (tailoring, adding new material or interventions that are conceptually consistent with the EBP, and changing the length of the session or protocol without removing core elements) to be fidelity-consistent. We determined those that involved alteration or non-delivery of core elements of the intervention (removing elements of the protocol, loosening the structured elements of the protocol, drift from the protocol, and integration of other established treatment modalities or strategies that are not consistent with the EBP's approach) to be fidelity-inconsistent. Other types of modification in the framework (e.g., adjusting the order intervention components or substitution of elements) might depend on the degree and content of the changes and were therefore not categorized as inherently fidelity consistent or inconsistent.

Emerging evidence indicates that clinicians make a variety of unplanned modifications when implementing EBPs in routine care, some of which are fidelity-consistent, and some of which are not. In a study of clinicians in a community mental health system, the most commonly endorsed modifications were tailoring the intervention to meet the needs of patients, drifting from the protocol, integrating other interventions with the EBP, and loosening the structure of the treatment protocol [11]. Similarly, clinicians on inpatient PTSD units endorsed a number of modifications, including tailoring the intervention, integration, and changing the length of the protocol [10]. Clinician interviews further indicated that some modifications made in usual care are inconsistent with the process of careful, measured modification that has been advocated for successful long-term implementation. Identifying factors associated with modifications that are inconsistent with intervention fidelity, current evidence, or planned adaptation can lead to a better understanding of how to increase the delivery of EBPs with adequate fidelity or planned and thoughtful modifications when necessary.

There is increasing evidence that clinician characteristics can uniquely influence the use of EBPs [18]. Several individual-level factors might influence clinician behavior related to modification, including patient population, training success, and provider attitudes toward EBPs. Interviews with clinicians indicate that some make modifications in response to contextual challenges or to address specific provider or client characteristics or needs that are not perceived to be sufficiently addressed in the standard protocols [9–11]. For example, clinicians who work with particular patient populations might find that they need to tailor the intervention to accommodate differences in developmental stage or cognitive ability [19]. Additionally, the success of prior training efforts may impact the tendency to modify EBPs. Clinicians who have better mastery of the EBP protocol may be better prepared to adapt interventions appropriately while maintaining fidelity to core treatment elements. They may also be more able to identify instances in which they are delivering or deviating from EBPs [20]. A link between provider attitudes and training outcomes has also been observed. Clinician attitudes toward EBPs prior to training, specifically clinicians’ openness to the use of EBPs, willingness to use EBPs if required to do so, or perception of their appeal, predict knowledge and fidelity following training [21, 22]. Furthermore, in a recent study in a public sector mental health setting, clinicians with greater openness were more likely to endorse the use of cognitive behavioral therapy with their child clients [18], while those who perceived greater divergence between their practice and EBPs were more likely to use psychodynamic psychotherapy techniques. Taken together, these findings suggest that attitudes may also be linked with use of and modifications to EBPs following training.

In the current study, we used follow-up data from two studies on EBP training and implementation to examine individual clinician-level factors associated with subsequently reported fidelity-consistent modifications and fidelity-inconsistent modifications among clinicians trained to deliver cognitive behavioral therapy (CBT) to child or adult populations. Based on previous literature [22], we hypothesized that training success, defined as meeting criteria for successful completion of an EBP training program, and EBP attitudes, specifically openness to using EBPs and willingness to adopt if the intervention had appeal, would be associated with more subsequent fidelity-consistent modifications (hypothesis 1). Second, we hypothesized that training success, appeal, and openness would be associated with subsequent fewer fidelity-inconsistent modifications and that perceived divergence of EBPs from clinicians’ routine practice would be associated with more subsequent fidelity-inconsistent modifications (hypothesis 2).

## Method

Participants were clinicians in the Philadelphia area who received workshop training followed by consultation in cognitive behavioral therapy for children or adults. In both samples, participation in training was intended to be voluntary, although in the adult sample, some clinicians indicated that they felt some pressure to participate by their agency administration [23]. The adult training included a workshop that covered basics of CBT, with applications for depression and commonly co-occurring problems including anxiety and substance abuse. The child training focused on child anxiety. The core principles of CBT that were conveyed in training across the two samples were consistent (e.g., psychoeducation, cognitive restructuring, behavioral experiments). Consultation for both samples included discussion of application to specific cases, didactic topics (such as treating clients with comorbid diagnoses), practice with concepts and interventions, and assistance in implementation of the treatment within the practice setting [24, 25]. In addition, fidelity was emphasized in both training programs through discussion of core elements of the treatments, feedback on delivery of core elements of treatment, and the use of fidelity assessment instruments. While participants were encouraged to self-assess their fidelity during training and after training was completed, it was not a requirement. A sample of clinicians who served adults (*n* = 27; 39 % of clinicians who received training) and children (*n* = 50; 43 % of clinicians who received training) agreed to participate in a study of their use of CBT following the training [11, 26] and were interviewed at a 2-year follow-up. For both studies, investigators had attempted to contact all participants in the initial training three times. One clinician in the adult sample declined participation and 12 had left their positions and did not provide contact information; 2 of them had left the field to pursue other careers. Two individuals in the adult sample initially agreed to participate in a follow-up interview but did not follow through with scheduling despite repeated efforts. The other non-participants did not respond to our requests for follow-up interviews.

### Adult sample

Twenty-seven providers who were trained in CBT for adults participated in interviews. Sixty-seven percent (*N* = 18) of them were female. Eighty-one percent (*N* = 21) had a master’s degree, 7 % (*N* = 2) had completed some graduate work (such as toward a master’s degree), and 12 % (*N* = 4) had a bachelor’s degree. Seventy percent (*N* = 19) of participants were Caucasian, 19 % (*N* = 5) were Black, and 4 % (*N* = 1) were Asian; 7% (*N* = 2) were also Latino, and 7 % (*N* = 2) were multiracial or endorsed a different race or ethnicity. Comparisons with the full sample of providers who were trained revealed no significant differences in groups in terms of demographics or educational background.

### Child sample

Fifty providers who were trained to provide CBT to children participated in interviews. Ninety-two percent (*N* = 46) of them were female. Eighteen percent (*N* = 9) had a doctoral degree, 65 % (*N* = 32) had a master’s degree, and 18 % were in graduate school working toward an advanced degree (*N* = 9). Seventy-four percent (*N* = 37) of participants were Caucasian, 8 % (*N* = 4) were African-American, 8 % (*N* = 4) were Asian American, 4 % (*N* = 2) identified as “other,” none identified as Hispanic/Latino, and ethnicity was missing for 6 % (*N* = 3) of the sample. Educational and demographic status of this sample was generally similar across the full sample that received training and the participants in this study, although fewer participants in this study identified as Hispanic/Latino [26].

## Measures and procedures

### Attitudes

#### Evidence-Based Practice Attitude Scale [27]

The Evidence-Based Practice Attitude Scale (EBPAS) was administered prior to training to measure therapist attitudes toward EBPs. It is a 15-item well-established measure of provider attitudes toward EBPs. Participants indicate on a four-point Likert-scale (0 = “not at all” to 4 “to a very great extent”), the extent to which they agree with a particular statement. Higher EBPAS total scale means scores indicate more favorable attitudes. The EBPAS contains four subscales, each of which has been associated with specific clinician characteristics and occupational settings. The Appeal scale represents the extent to which the provider would adopt a new practice if they believe it is intuitively appealing, makes sense, could be used correctly, or is being used by colleagues who are happy with it. The Openness scale represents the extent to which the provider is generally open to trying new interventions or would be willing to try or use new types of therapy. The Divergence scale score is the extent to which the provider perceives research-based interventions as different from their own practice, not clinically useful, and less important than clinical experience. The Requirements scale reflects an inclination to comply with requirements to adopt a new practice enacted by an agency, supervisor, or state. Prior research on the measure’s psychometric properties demonstrated good internal consistency for the EBPAS scales, with Cronbach’s alphas of 0.76 for the total scale and 0.66 to 0.91 for the subscales [28].

### Training outcomes

Criteria for successful training for each training program were used to represent training outcomes. For each sample, clinician work samples were assessed using the fidelity rating systems that were developed to evaluate fidelity in clinical trials for the interventions [29, 30]. Clinicians whose competence scores reached the benchmark scores required for clinicians in clinical trials were considered successfully trained.

#### Adherence and Skill Checklist [31]

The Adherence and Skill Checklist (ASCL) was used to assess fidelity for the sample trained to deliver CBT for children. This instrument measures (a) adherence to the content of CBT for child anxiety and (b) skill or competence in treatment delivery. The ASCL measured adherence and skill demonstrated in 8-min performance-based behavioral rehearsals [15], which involved clinicians preparing an anxious child (played by a trained undergraduate) for an exposure task. Inter-rater reliability for the total adherence score was an ICC of 0.98; for skill, the ICC was 0.92. As a validity check, experienced CBT therapists reviewed the ASCL and rated it as accurately capturing the components of CBT for youth anxiety. To be considered successfully trained to criterion, the criterion for inclusion as a therapist in a clinical trial was defined as being rated as a 5 or higher on a 6 point Likert scale (i.e., 80%) on skill in applying CBT.

#### The Cognitive Therapy Rating Scale [32]

The Cognitive Therapy Rating Scale (CTRS) was used to assess fidelity for the sample trained to deliver CBT to adults. The CTRS is an 11-item scale that measures cognitive behavioral therapist competence. Expert raters evaluated a complete session and assessed general therapeutic skills, the therapist’s ability to structure the session, and ability to intervene using the most appropriate CBT methods. Beck Initiative training consultants received training in conducting the ratings using a standard set of recordings and accompanying ratings and attended monthly meetings in which a session was rated and discussed by all training consultants to ensure consistency in rating. Per-judge reliability was assessed periodically. The intra-class correlation for the 15 sessions rated by all training consultants was 0.61, indicating good agreement [33]. The convention for CBT clinical trials (CTRS total score ≥40; [34]) was used as a threshold to indicate, and raters agreed about achievement of this standard for 93 % of the 15 sessions that they rated together. To be considered successfully trained, clinicians needed to achieve this standard on a recorded session with one of their clients by the end of training.

### Modification

#### Follow-up interview on the use of and modifications to CBT [11]

Therapists in both samples were interviewed using the same standard interview questions about their use and adaptation of CT 2 years after they completed the training and consultation. Therapists were asked to describe adaptations that they had made to the protocol as a matter of routine or for specific patients. Follow-up questions were used to probe for additional information. The interviews were coded separately for each sample using a framework and coding system of modifications and adaptations made to EBPs [16]. This framework included 12 codes, definitions, and decision rules for identifying types of modifications made to the content of the treatment, including tailoring the intervention (making small changes to fit patient needs without altering the intervention content), adding or removing components, lengthening or shortening the protocol or sessions, integration of other intervention into the EBP or integration of EBP components into other interventions, changing the sequence of interventions, and drifting from the protocol. The first author trained all raters on the use of the coding system until they achieved 90 % agreement. Interviews were reviewed and divided into separate segments for each modification that a clinician described. Raters coded them for the presence or absence of each content-level modification. Rater agreement for both samples was high, ranging from *k* = 0.80 for the adult sample and k = 0.98 for the child sample.

### Data analytic strategy

Because prior research, including a larger study that included our CBT for children training sample, demonstrated a relationship between attitudes and training outcomes and because the clinicians in our sample participated in two different training programs, we first examined whether pre-training attitudes or participation in training for CBT for children vs. adults were associated with training success. To assess the unique variance in the association with subsequent training success (being trained to criteria for competence) for each theoretically relevant variable that we planned to use in subsequent analyses, a logistic regression was conducted, with a variable indicating the sample/training program (therapists who were trained provide CBT to adults vs. those who were trained to provide CBT to children) and each EBPAS subscale entered into the model.

To examine our hypotheses regarding modification, the number of each type of modification (fidelity-consistent and fidelity-inconsistent) that clinicians endorsed or described in their interviews was totaled, and these totals were standardized so that the data were normally distributed. To evaluate our hypotheses that training success or attitudes toward EBPs would be associated with subsequent fidelity-consistent or fidelity-inconsistent modifications, hierarchical linear regressions were conducted. The regression analyses examined whether training success and provider attitudes were uniquely associated with these modifications after controlling for whether the provider delivered psychotherapy to children or adults. Two separate hierarchical regressions were run to examine the proposed hypotheses about modifications. For these analyses, the fidelity-consistent modifications (hypothesis 1) or fidelity-inconsistent modifications (hypothesis 2) were entered as the dependent variable. For the dependent variables, a dichotomous variable representing whether participants were trained to provide CBT for children vs. adults was entered into Step 1 to control for differences in the populations and protocols, and each EBPAS subscale and a dichotomous variable indicating whether or not each clinician was trained to criterion was entered into Step 2. To assess for multicollinearity, tolerance and variance inflated factor statistics (VIFs) were examined, and all VIFs were 2 or below. Partial correlation coefficients were calculated to assess the incremental effect of each variable in the models. An a priori sample size calculation determined that our sample size was adequate to detect an effect (Cohen’s *f*^2^) of 0.20 or higher (a small-to-medium effect) using the specified models at a power level of 0.80.

## Results

### Sample characteristics

Comparisons between clinicians who were trained in CBT for children vs. adults indicated no differences in terms of minority status (*χ*^2^(1, *N* = 77) = 0.620, *p* = 0.431). Clinicians who served adults were more likely to be male (*χ*^2^(1, *N* = 77) = 6.86, *p* = 0.009) and less likely to endorse a primarily CBT orientation prior to training (*χ*^2^(1, *N* = 77) = 15.24, *p* < 0.001). The proportion of clinicians possessing an advanced degree did not differ between groups (*χ*^2^(1, *N* = 77) = 0.199, *p* = 0.650).

### Training success, baseline attitudes, and factors associated with subsequent modifications

Receiving the training to deliver CBT to adults was significantly associated with being trained to criterion, *χ*^2^ (1, *N* = 70) = 4.86, *p* = 0.028; odds ratio (OR) = 0.21; 95 % confidence interval (CI) (0.05–0.85). Among clinicians in the CBT for adult training, 71 % achieved criterion for competence, and among the clinicians trained in CBT for children, 60 % achieved competence. Contrary to findings from previous research, in this sample, attitudes toward EBPs, as assessed at baseline, were not associated with subsequent training success: Appeal *χ*^2^ (1, *N* = 70) = 2.08, *p* = 0.15; OR = 0.52; 95 % CI (0.21, 1.27); Openness *χ*^2^ (1, *N* = 70) = 0.38, *p* = 0.54; OR = 1.34; 95 % CI (0.53, 3.35); Divergence *χ*^2^ (1, *N* = 70) = 0.56, *p* = 0.46; OR = 0.70; 95 % CI (0.27; 1.79); Requirements *χ*^2^ (1, *N* = 70) = 1.59, *p* = 0.20; OR = 1.34; 95 % CI (0.85, 2.01). Table 1 contains descriptive statistics for modifications and the EBPAS scales, which were similar to those of a sample of community-based therapists who received training in a previous study [22]. Regression analyses assessing differences between training cohort and attitudes revealed that adult providers endorsed greater divergence between EBPs and their routine practice (*F* = 16.54; *p* = 0.000; *R*^2^ = 0.208), but no differences were identified for other subscales. Table 2 presents correlations between the variables that were investigated. Table 3 contains the results of hierarchical linear regression analyses conducted to examine factors associated with each type of modification.

**Table 1:** Descriptive statistics

	Mean	SD	Range
Adaptations			
Fidelity consistent	1.14	1.65	0–7
Fidelity inconsistent	3.34	2.62	0–10
EBPAS scales			
EBPAS appeal	3.33	0.73	0.25–4
EBPAS openness	3.19	0.63	1.25–4
EBPAS divergence	1.10	0.74	0–3
EBPAS requirements	2.85	1.34	0.01–7

Note: values for modifications were normalized prior to regression analyses

**Table 2:** EBPAS correlations table

Scales	EBPAS requirements	EBPAS appeal	EBPAS openness	EBPAS divergence
EBPAS requirements	1			
EBPAS appeal	0.31*	1		
EBPAS openness	0.19	0.35**	1	
EBPAS divergence	−0.02	−0.31*	−0.26*	1

*EBPAS* Evidence-Based Practice Attitudes Scale

*p < 0.05; **p < 0.01

**Table 3:** Hierarchical regression analyses

	Number of fidelity consistent modifications			Number of fidelity inconsistent modifications		
Variable	R^2^	β	Partial correlation	*R* ^2^	β	Partial correlation
Step 1	0.14			07		
Adult vs. child		−0.37**	−0.37		−0.27*	−0.27
Step 2	0.29			24		
Adult vs. child		−0.29*	−0.28		−0.19	−0.18
EBPAS appeal		0.15	0.16		0. 31*	−0.29
EBPAS openness		0.24***	0.26		−0.08	−0.08
EBPAS divergence		0.10	0.10		0.04	0.04
EBPAS requirements		0.16	0.17		−0.20	−0.20
Trained to competence criteria		0.07	0.08		0.36**	0.36

*p < 0.05; **p ≤ 0.01; ***p = 0.051

#### Hypothesis 1: fidelity-consistent modifications

After controlling for whether clinicians were trained in CBT for adults vs. children in the first block, contrary to our hypothesis, being trained to criterion was not associated with subsequent fidelity-consistent modifications. Clinicians who were trained in CBT for adults were more likely to make fidelity-consistent modifications. Our hypotheses regarding baseline clinician attitudes were largely unsupported. Higher scores on the openness scale were associated with subsequent fidelity-consistent modifications at a marginally significant level (*p* = 0.051), suggesting that clinicians who endorsed greater openness to trying new interventions may be more likely to make modifications that preserved core elements of CBT. However, contrary to our hypothesis, scores on the appeal scale were not associated with fidelity-consistent modifications.

#### Hypothesis 2: fidelity-inconsistent modifications

An identical model was used to examine fidelity-inconsistent modification as the dependent variable. Training success was associated with subsequent fidelity-inconsistent modifications, but, contrary to our hypothesis, clinicians who were trained to criterion reported more fidelity-inconsistent modifications. The EBPAS appeal scale was significantly associated with subsequent fidelity-inconsistent modifications, such that clinicians with higher appeal scores made more fidelity-inconsistent modifications. However, the openness and divergence scales were not associated with fidelity-inconsistent modifications at follow-up.

## Discussion

Implementation frameworks suggest that modifications should be carefully considered and, if made in the absence of empirical support, that their impact should be measured [6, 12, 35, 36]. Evidence and practical implementation experience is mounting to suggest that clinicians modify EBPs in community mental health settings but that these modifications do not appear to be consistent with published definitions of adaptation, as they are not made in conjunction with a process of using research or practice-level data to determine their necessity or to examine their impact [9–11]. Importantly, there is a dearth of literature around identification of modifiable predictors of fidelity-inconsistent modifications, which have either been associated with poorer clinical outcomes in previous research or have not improved clinical outcomes [34, 35, 37–39]. This study contributes to the literature by examining this important question in a sample of clinicians who were trained to provide CBT to adults or children in an urban mental health system. We examined whether individual-level clinician factors, including training success and attitudes about EBPs, were related to subsequent modification of EBPs. Training success was not associated with subsequent fidelity-consistent modifications, but it was associated with fidelity-inconsistent modifications in an unexpected direction. The EBPAS appeal scale, which indicates the extent to which clinicians endorsed willingness to adopt EBPs if they found them to be intuitively appealing, believed they could be used correctly, or had colleagues who used them successfully, was uniquely associated with subsequent fidelity-inconsistent modifications, and openness to the use of EBPs was associated with fidelity-consistent modifications.

Being trained to criteria for competence in delivering CBT emerged as an important factor associated with clinician reports of fidelity-inconsistent modifications, contributing the greatest unique variance to the model we tested. Although our data do not allow us to make a definitive conclusion, a potential explanation is that clinicians who attained the competencies necessary to implement CBT were better able to recognize when they were departing from the protocol. It is important to note that in this sample, the reported number of modification types was relatively low. It is possible that clinicians underreport modifications (i.e., modify in more ways than they report doing so) and that clinicians who are trained to criterion have a more complete and comprehensive recognition of what is consistent with the protocol (cf., [22]). If this were the case, they may be more able to report accurately when they make modifications that are inconsistent with the protocol. Because clinicians were asked to describe the *types* of modifications and adaptations that they make in practice, rather than provide an accounting of how many modifications they make in a particular period of time, our findings suggest that clinicians may gravitate toward relatively few specific modifications rather than making a wider variety of modifications or that specific modifications are more salient and easier to recall or identify than others they made have made. Previous research has also indicated that therapists can provide accurate reports of therapeutic practices they deliver but that more training and support may be needed to improve their ability to report more subtle aspects of implementation [20]. In light of this previous research, our preliminary findings encourage further research to better understand the relationship between training and clinician report of modifications.

Some clinician attitudes that have been identified as individual-level predictors of training outcomes and implementation (e.g., [21, 22]) were also found to be associated with modifications to CBT in this study. Clinicians who were open to using evidence-based practices were more likely to make fidelity-consistent modifications, suggesting that clinicians who are open to EBPs may be more likely to try to find ways to make them work in routine care while delivering the intervention as closely to the protocol as possible. Baseline scores on the EBPAS appeal scale, which assesses whether willingness to adopt EBPs is contingent on finding an intervention appealing or having positive experiences with it, were associated with fidelity-inconsistent modifications at follow-up. This finding raises the possibility that clinicians who made more fidelity-inconsistent modifications may have had less positive experiences with CBT or found it less appealing when actually implementing it, and may therefore have seen less value in maintaining fidelity. While these findings are not entirely consistent with other studies that have identified either no association [40], or a positive relationship between clinician attitudes and training outcomes [22], differences in strategies for assessing use of and fidelity to EBPs may account for these discrepancies. Taken together, though, our results reinforce previous findings indicating that clinicians may need to have favorable attitudes toward EBPs to deliver them with fidelity [41]. Collectively, these studies indicate that training is a clear target for dissemination and implementation efforts to focus upon. In particular, ensuring that therapists are trained to deliver EBPs with fidelity and able to identify modifications that are consistent and inconsistent with fidelity while considering their impact on clinical outcomes may result in higher fidelity or more planful adaptation. It may also be important to provide specific guidance around adaptation and modifications as a component of training and consultation programs, and to identify ability to utilize different data sources to guide the process of adaptation as a competency goal for training. Furthermore, training programs may target clinician attitudes by presenting compelling empirical and experiential evidence of benefits to adoption and providing support around overcoming challenges to implementation at the individual level. Finally, training in and possibly requirement of ongoing fidelity monitoring and the use of outcome monitoring may improve fidelity or facilitate planned adaptation while reducing fidelity-inconsistent modifications.

Although this study has a number of strengths, namely being the first prospective study to focus on individual-level factors associated with subsequent modifications to EBPs, there are limitations. First, we relied on interviews to obtain information about the types of modifications that clinicians make in usual care, introducing the potential for recall bias or failure to recognize specific modifications in their own practice. It is likely that modification is better captured when observed in audiotaped sessions by expert raters and in conjunction with fidelity ratings [6]. It will be important in future research to objectively identify modifications, assess reasons for making specific changes, and to examine the impact of the modifications on clinical outcomes. Second, while organizational-level factors may also influence decisions to modify EBPs, our sample size precluded the introduction of additional variables into our model. Third, although roughly comparable, we combined two samples that participated in different training programs, which could have resulted in cohort effects. Furthermore, while the core interventions of the two CBT treatments are very similar, some of the differences in modifications found at the child vs. adult level may also have been accounted for by differences in the populations or aspects of the protocols. While we did control for some of these differences in our regression models, additional research to replicate these findings will be necessary, particularly with sample sizes that are adequate for an exploration of potential interactions between cohorts, attitudes, and modifications. Finally, the clinicians who participated in this study were only a subgroup of all clinicians who received training, although comparisons revealed very few differences between the full sample of trained clinicians and those who participated in the follow-up study.

There is little doubt that clinicians modify EBPs in routine care settings. However, there are potential implications of modifying EBPs, and these are the subject of considerable debate in the literature [6, 35]. Possibilities include improved clinical outcomes and sustainment of EBPs in routine care [35], but there is also the potential for decreased fidelity to essential treatment elements and poorer treatment outcomes [42]. Rather than approaching the modification of EBPs as uniformly positive or undesirable, it is important to empirically and conceptually differentiate between fidelity-consistent and fidelity-inconsistent modifications and planned adaptations. To fully understand their impact, improved measurement of different types of modifications (e.g., via observation) and monitoring of outcomes must occur. Future research is also necessary to elucidate whether specific modifications or planned adaptation result in a “voltage drop” [35] or in similar or even enhanced outcomes for clients. Studies are underway to shed light on this important question, and practice-based research can further this goal [35, 43].

Until more is known about the ways in which modifications impact clinical outcomes in different contexts, developers of complex psychosocial interventions, implementation facilitators, and training consultants can either allow modifications to occur naturally and hope for good outcomes or attempt to facilitate an effective process of adaptation. The development and testing of highly flexible protocols that include guidance on selection of appropriate modules or interventions is a promising direction toward this goal [44]. The results of this study also suggest that training programs should focus on training clinicians to competence, fostering positive attitudes and experiences with EBPs, and facilitating a thoughtful process of outcomes monitoring to guide decision-making and adaptation [35]. In-depth discussions about the relationship between fidelity and modifications, along with ongoing consultation to provide assistance around appropriate adaptation to core and peripheral elements can further enhance implementation efforts.
